# Understanding Generational Differences in Muslim American Mental Health Help-Seeking

**DOI:** 10.1177/13634615261471970

**Published:** 2026-08-13

**Authors:** Leena Badran, Niveen Rizkalla, Steven P. Segal

**Affiliations:** 1 School of Social Welfare, 8789 University of California Berkeley, USA; 2 Department of Psychiatry and Behavioral Science at the University of California, USA; 3 Center for Effective Global Action (CEGA) and The School of Public Health, 1438 University of California Berkeley, USA

**Keywords:** generational gaps, intentions, Muslims, pathways, religion, seeking mental health help

## Abstract

Attitudes toward mental health help-seeking are shaped by a complex interplay of environmental, sociocultural, religious, and generational factors. Within Muslim communities, these attitudes are further informed by religious teachings, cultural norms, and intergenerational dynamics. This study explores the disparities in mental health-seeking behavior between first- and second-generation Muslim Americans. The sample consisted of 37 participants, including 16 first-generation immigrants (foreign-born) and 21 second-generation individuals born in the USA to at least one immigrant parent. The study used a combined deductive and inductive thematic approach to the analysis of semi-structured interviews. Interview content codes and themes were developed using a six-phase method for qualitative thematic analysis. Four major themes emerged encompassing distinct subthemes. First, acculturation effects including: (a) mental health awareness, normalization, and knowledge; and (b) the influence of acculturation on attitudes. Second, intentions including: (a) first-generation participants often sought private help-seeking; and (b) openness to public help-seeking and a health-conscious orientation. Third, perceived behavioral control. Fourth, pathways to seeking mental health care including: (a) preference for informal support systems; (b) incorporation of religious aspects; and (c) a preference for Muslim therapists. The first three themes highlighted notable intergenerational differences; the fourth theme revealed shared pathways to seeking care between the two groups. Culturally adapted practices to mental health service provision should integrate both traditional and non-traditional cultural awareness while addressing intergenerational gaps to enhance mental health support within these communities.

Scholarly work reveals that attitudes and service utilization for mental health vary across ethno-racial and religious communities. Studies found that minority groups, including Muslims in the USA, are less likely to seek help for mental health challenges despite coping with such challenges in high rates (Al-Krenawi & Graham, 2011; Amri & Bemak, 2013). This reluctance to use formal mental health services persists even though timely interventions can prevent the progression of psychological or psychiatric issues into chronic conditions (Christodoulou & Christodoulou, 2007).

Gaps between generations can occur regardless of the society or culture in which individuals reside. Social processes such as globalization, technology, mobility, historical periods, life expectancy, changes in family structure, and migration are some of the factors contributing to the disparities between generations (Al-Lawati, 2019). The intergenerational gap in attitudes toward seeking mental health help presents challenges in both understanding and addressing mental health needs within Muslim communities in the USA (Mudryk & Johnson, 2023). Although Muslim young generations are more open to integrating professional mental health services into their lives when required (Aromaa et al., 2010), older generations often retain traditional spiritual solutions and support within the family in a time of need, reflecting the broader societal stigma surrounding mental illness (Jimenez et al., 2012, 2022; Mudryk & Johnson, 2023). In general, it has been observed that the younger Muslim generation who were offspring of immigrants and natives of eastern cultures, embraced seeking mental health help accustomed to western countries/cultures where they were born and raised, while also preserving the eastern cultural values rooted in their parents’ heritage. This duality may lead to a cognitive dissonance between the two generational perspectives, causing conflicts and distress (Ahmed, 2019; Rasmi et al., 2015). This divergence can create barriers to effective communication and deprive support within families, which might ultimately affect the well-being of individuals and the community (Herzig, 2011).

## Muslims in the USA

The Muslim American population is diverse and rapidly growing, with an estimated 3.45 million individuals of all ages, of which 2.15 million are adults. This population is characterized by a mix of backgrounds/nationalities among immigrants and their descendants, and is divided into three main groups: first-generation (58%), immigrants who were born in another country; second-generation (18%), citizens who were born in the USA with at least one immigrant parent; and a third group of approximately 24% who are US natives with both parents born in the USA (Pew Research Center, 2017).

The Muslim population in the USA faces a variety of social, cultural, and political challenges, leading to heightened stressors. Among these various stressors are cultural adjustment, immigration difficulties, language barriers, discrimination, and Islamophobia, which have been strongly linked to negative mental health impacts (Al Wekhian, 2015). Existing studies point to a general reluctance among Muslims to engage with mental health services due to multiple factors (W. Abu-Ras, 2007; Aloud & Rathur, 2009; Martin, 2014).

Muslims live within a strong family system embedded in a collectivistic society, where social influences from family, peers, and religious scholars and institutions significantly impact daily decision-making. In addition, Islam encourages collectivism as a way of life among Muslims (A. Ali et al., 2020). Muslim families often maintain collectivist values and norms, in perceiving an individual's crisis as part of a family concern that affects the entire system, including the broader community (Christodoulou & Christodoulou, 2007). Therefore, collectivism might promote a preference for seeking mental health support from informal sources within the family or community, which at times is compounded by a significant social stigma surrounding mental health conditions (Badran et al., 2025a, 2025b). Imams, as Muslim faith leaders, play a crucial role in supporting community mental health. Although Muslim Americans may be inclined to seek guidance from religious leaders because of cultural familiarity, mistrust of formal systems, or concerns about social stigma, engagement with Imams should not be viewed as mutually exclusive from professional mental health care. Recent literature highlights the potential for faith leaders, including Imams, to serve as effective bridges to mental health services by providing initial support, reducing stigma, and facilitating appropriate referrals to professional care (Alhomaizi et al., 2018; Ciftci et al., 2013; Humam et al., 2023).

Muslim Americans often integrate aspects of the biopsychosocial model when understanding mental illness. This model is based on the idea that mental illness results from the interplay between a person's biology, internal mental experiences, and social and experiential factors, including religion and spirituality, that shape these experiences. By contrast, the predominant western biomedical model primarily frames mental illness as a medical condition rooted in biological dysfunction and typically emphasizes diagnosis, pharmacological treatment, and clinical intervention (Roberts, 2023). Although they typically adhere to the predominant western biomedical model, many also perceive mental illness as God's will, a test or punishment, or even possession by spirits, perceiving it as an opportunity to strengthen their connection with God (Ali et al., 2022). Findings indicated that the biomedical beliefs and Islamic religiousness predicted more openness to mental health care. Such a finding offers a nuanced understanding of how biological aspects of mental health may align with Islamic spiritual values (Mudryk & Johnson, 2023).

Religion holds a significant value for Muslims, not only for its spiritual attributions, but also as a coping mechanism. The role of religion as a protective factor has been examined in various studies, yielding mixed results (W. Abu-Ras & Abu-Bader, 2009). However, there is empirical support for religiosity and religious coping serving as protective factors against psychological distress (Goforth et al., 2014; Ikizler & Szymanski, 2018; Rizkalla et al., Under review). Middle Eastern/North African Americans turn to religion for comfort, particularly if they attribute their distress to supernatural causes or if they have strong beliefs in religious fatalism. For these individuals, religious beliefs provide solace and a way to make sense of their struggles (Aloud & Rathur, 2009).

The interplay between the multiple identities Muslim Americans hold—religious, ethnic, national—and belonging can heighten their stress levels, especially among immigrants (Berry, 2005; Han & Pong, 2015; Kaur, 2022; Saechao et al., 2012). Immigrants encounter various degrees of acculturation stress based on factors such as reasons for immigration, adjustment to the western country/society, English proficiency, sociopolitical history, socioeconomic status, experiences of trauma, and racial or ethnic discrimination (Amri & Bemak, 2013). Herein, we try to understand how individuals of different generations have integrated these challenges and demands associated with seeking mental health service following the theory of planned behavior. This theory explains behavior by focusing on three main factors: attitudes, subjective norms, and perceived behavioral control. Attitudes refer to an individual's personal views and feelings about a behavior and whether they see it as positive or negative. Subjective norms describe how individuals perceive social pressure, including whether people important to them approve or disapprove of the behavior. Perceived behavioral control reflects how capable a person feels in performing the behavior, taking into account their own abilities as well as any external barriers they may face (Ajzen, 1991). This paper explores the intergenerational gaps in attitudes toward seeking mental health help within Muslim communities in California, considering the cultural, religious, and societal factors that contribute to these disparities. Understanding these gaps might assist in developing sensitive and culturally appropriate mental health interventions that cater to the needs of all generations in this unique minority group.

## Methods

### Participants and Procedure

Thirty-seven American Muslims who reside in California, USA participated in the study (10 men and 27 women). The participants’ ages ranged from 18 to 63 (*M* = 30, *SD* = 13), 78% described themselves as religious and 27% were students. Immigration classification was determined as follows: 16 participants were first-generation immigrants and 21 were second-generation immigrants. Participants from both groups had diverse ethnic backgrounds and originated from the following regions: North Africa, South Asia, Europe, Southeast Asia, Middle East, and West Africa (Table 1).

**Table 1.** Sample characteristics

		First generation (*n* = 16)	Second generation (*n* = 21)
		43%	57%
Gender	Men	6	4
	Women	10	17
Level of education	BA	6	6
	MA	6	3
	PhD	2	2
	Student	0	10
	Other	2	0
Marital status	Single	0	16
	Married	16	5
Work in the Mental health sector	Yes	1	1
	No	15	20
Northern and Southern California	Northern	12	17
	Southern	4	4
Level of religiosity	Very religious	1	5
	Religious	10	13
	Traditional	5	2
	Secular	0	1
Economic status	High	4	0
	Moderate	11	15
	Low	1	6
History of seeking mental help from a therapist/psychologist	Yes	4	8
	No	12	12
	Not sure	0	1
History of psychiatric counseling	Yes	0	2
	No	16	18
	Not sure	0	1
Diagnosed with a mental health issue*	Yes	0	4
	No	16	17
Age	Min	28	17
	Max	63	32
	Mean	43 ± 9	21 ± 5

*Diagnosis of metal health issues included schizophrenia, anxiety, and post-traumatic stress disorder.

### Procedure

The study received approval from the Institutional Review Board at the University of California, Berkeley (2022-12-15929). A convenience sampling method was used, with the researchers (first two authors) reaching out to Muslim community centers, educational institutions (schools and universities), and various Muslim professional groups for recruitment. Initially, the researchers sought assistance in the recruitment of participants from Imams of mosques, project coordinators, and other community leaders. In instances in which collaboration yielded low responsiveness, snowball sampling was implemented. Inclusion criteria comprised: being a Muslim residing in California, at least 18 years old, and proficient in English or Arabic. Participant recruitment concluded upon reaching data saturation (Saunders et al., 2018) indicating that further interviews were unlikely to yield new information.

### Instrument and Data Collection

This qualitative study utilized semi-structured interviews for data gathering. Interviews lasted around 90 minutes and were by the first author in English or Arabic (based on the participant's preference) through the Zoom platform. Interviews were audio-recorded after gaining permission from participants who were only required to verbally consent to participate in the study. The co-authors are an interdisciplinary group of three researchers consisting of a Muslim Palestinian, a Christian Palestinian, and an American Jew, all of whom have studied the topic of mental illness among minorities. Guided by the theory of planned behavior (Ajzen, 1991), the interview guide was designed to explore participants’ mental health help-seeking intentions through three core constructs: attitudes, subjective norms, and perceived behavioral control. The guide consisted of ten open-ended questions aligned with these constructs to allow participants to describe their perspectives in their own words. The interview guide was reviewed by mental health experts serving Muslim and Arab communities and was refined based on their feedback.

### Data Analysis

Using a combined deductive and inductive thematic approach within the areas of the theory of planned behavior, the data was systematically coded to extract themes based on participants’ responses. The codes and themes were developed using Braun and Clarke (2006) six-phase method for qualitative thematic analysis. The audio-recorded interviews were initially transcribed and translated by the first author. The transcripts were then analyzed by both the first and second authors, who were responsible for reviewing all the transcripts to create initial codes and organize the data to identify themes. Major themes were inductively identified from these codes. Following this, the first two authors reviewed and categorized the coded data into major themes and subthemes, ensuring that these were well-supported by the transcripts (Smith, 2003). Regular meetings among the authors facilitated the comparison and refinement of themes and subthemes, with any disagreements resolved through discussion until a consensus was reached. This collaborative process clarified and re-defined the themes and refined their organization and interrelationships. Given the research team's insider perspectives, reflexivity was practiced throughout the analytic process. The researchers engaged in ongoing self-reflection, discussed assumptions during team meetings, and used iterative coding and peer debriefing to reduce potential bias and maintain interpretations closely grounded in participants’ narratives. Hard and electronic copies of the transcripts were used during the analysis, which was conducted manually, instead of using specialized software. To maintain participant anonymity and confidentiality, pseudonyms were used. The third author supervised the study design, ethical and institutional procedures, manuscript preparation, and reviewed and edited the final version of the manuscript.

## Results

Our analysis revealed four main themes with several subthemes. First, acculturation effect: (a) mental health awareness, normalization, and knowledge; (b) acculturation impact on attitudes. Second, intentions: the first-generation participants often sought mental help secretly, whereas the second-generation participants were inclined toward being more open and health-conscious oriented. Third, perceived behavioral control. Fourth, pathways to seeking mental health care: (a) preference for informal support systems, (b) incorporation of religious aspects in seeking help, and (c) preference for Muslim therapists.

We have organized the analysis of themes based on intergenerational differences and similarities.

### Differences Between Generations

#### Acculturation Effect

##### Mental Health Awareness, Normalization, and Knowledge

The second-generation group expressed greater awareness and normalization of mental health issues and felt more at ease to voice their need to seek help compared with the first-generation group. As Amna said: “I’m a millennial so I think we’ve normalized mental health issues and seeking professional mental help” (woman, 23 years, Middle East, second generation).

Although still leaning toward traditional values, participants from the second generation have been exposed to the normalization of mental health care in California through schools, higher education, employment, and media.

The younger generation, they go to school or college, they have non-Muslim friends, they’re open to everything. They know more and know how to access more help. It is at schools mostly. You can seek the counselors for help. You can ask a friend who knows a person. It's like a domino effect, somebody knows somebody and help is everywhere. (Man, 22 years, Middle East, second generation )

By contrast, the first-generation participants found themselves still caught between the traditional cultural values they grew up with and those from the dominant culture. Soha mentioned that despite being exposed to western values and knowledge through various channels in the USA, it remains challenging for her to detach herself from her cultural roots:

Although we have studied and advanced in many areas here in America, we still cannot completely free ourselves from these (negative) beliefs about seeking psychological help. But within ourselves, we are aware and convinced that this is wrong and that we must see a psychologist or psychiatrist to help us. (Woman, 40 years, North Africa, first generation)

While contemplating mental health knowledge, most participants attributed mental health conditions to the biopsychosocial factors. However, there were participants from the first-generation group who associated mental health issues with religious explanations like weakness of faith.

The primary contributors to mental health issues are environmental factors such as social media, including peer pressure. The lifestyle prevalent in the West, along with materialistic influences, has a considerable effect on teenagers here and can lead to mental health issues. If you have strong beliefs, then these things [mental conditions] would not or should not affect you. (Man, 59 years, Middle East, first generation)

For Amna, mental health conditions may arise from different factors:

It might be trauma or chemical imbalances in the brain and for different conditions you need different professionals and treatments, deep depression where they can’t get out of bed so they’re going to see a psychiatrist because therapist wouldn’t really help you coach through. (Woman, 23 years, Middle East, second generation)

##### Acculturation Impact on Attitudes

Acculturation stress was observed among the first-generation participants through the spectrum of their beliefs and attitudes toward mental health. The second-generation participants described more benefits for seeking mental help support compared to the first-generation group (improvement of psychological and physical health, symptom management, social support, enhanced guidance that can prevent future complications). As Yasmeen said:

They may think what if someone finds out about my daughter, don’t think something is wrong with my household or with my upbringing … and I think because of that they may not either approach a mental health specialist or if they do, then they hide it from people. I also think that most don’t promote mental health support, and there is a lot of reliance on Imams. And we need more Muslim counselors who have the religious knowledge, but also can guide people on the emotional side. (Woman, 48 years, South Asia, first generation)

Unlike the first generation, participants who were from the second generation had more favorable attitudes toward seeking mental health help and considered it as equivalent to physical health.

[M]ental health is so important, and it really has to be in the right headspace at all times, no matter your background, no matter how old you are, or what you’re doing, it is exactly equal to our physical health. (Woman, 18 years, South Asia, second generation)

Waseem expressed confidence in his mental strength, reasoning abilities, and emotional self-control, framing psychological distress as a normal and manageable experience. By emphasizing his strong personality and perceived ability to cope independently, he articulated a rationale for why he did not view mental health services as necessary, stating:

We all get irritated and get tense and snappy sometimes, but I think I manage well. I don’t feel at any point that I personally need to seek any mental health assistance, I will not utilize it. (Man, 47 years, West Africa, first generation)

Waseem's framing reflects a psychological coping mechanism of rationalization, which may contribute to his avoidance of seeking professional mental health support.

#### Intentions

When both generational groups intended to seek help, the first generation did so secretly, striving to liberate themselves from cultural norms and social stigma. For the second generation, however, seeking mental health help was considered just one of many choices. Soha, who felt restrained by social and cultural stressors testified:

As I mentioned earlier, I can do that [seeking mental health help], but secretly, because of cultural and societal dependencies and societal pressures, it still scares me. I know it's wrong, but I can’t break free from it. (Woman, 40 years, North Africa, first generation)

Second-generation participants who sought mental health framed it as beneficial for their well-being rather than as a medical or abnormal issue:

I do talk to a therapist consistently, but that's mostly for like personal growth. (Woman, 23 years, Middle East, second generation)

#### Perceived Behavioral Control

Participants highlighted financial considerations as an important factor influencing access to formal mental health services. Second-generation participants who were mostly single, often relied on their parents to cover the cost of mental health sessions.

It's really expensive if you can’t use your family's insurance. Sometimes it's almost $200 an hour, which is just impossible for a student. (Woman, 20 years, South Asia, second generation)

By contrast, first-generation participants who were all married, described how marital and family status shaped perceived behavioral control, including decision-making and support within the household when considering seeking mental health care.

I would consult with my husband, my family, in case I feel depression and I feel that I need to see a psychiatrist. When he says ok to me, it will encourage me more to attend such help, but when he says no, and my situation is not a mental health issue then I may read Quran or Roqia for example and by these things I will get relief. (Woman, 45 years, Middle East, first generation)

### Similarities

#### Pathways to Seeking Mental Health Care

##### Preference for Informal Support Systems

Both groups prioritized informal support, with family as the preferred source of mental health help. The Muslim family remained central across generations. As Mesk said:

My parents play an important role in my life, and maybe my grandma and some other elders, but mostly it is my parents, especially when it comes to making decisions. (Woman, 19 years, Middle East, second generation)

Seeking mental health support within close circles was prioritized among participants, and only when no improvement was observed did they turn to external sources.

I have a sister here in America. I thought she really needed somebody professional. We tried all the things, we’ve been doing like as a religious family, as Muslims and Arabs, cultural things that didn’t work with her, so I advised her to go and see somebody professional. All the things we’ve been doing as religious, and as Muslims, they didn’t work with her, I advised her to go see a mental health professional. You reach a point when you can’t control it. (Woman, 19 years, South Asia, first generation)

##### Incorporation of Religious Aspects in Seeking Help

Leaning on informal resources for support was often coupled with the reliance on religious guidance, including consulting with an imam or engaging in pertinent religious practices, before considering formal mental health services. The emphasis of some participants on collective well-being could have led them to prioritize community-based solutions over individual therapy.

My parents would probably recommend it [relying on faith] as well, I think we are strong believers that the Quran can ease our pain, and when we can’t do it ourselves, we could go to a scholar that would help us. I do find it [helpful] sometimes when I'm down that listening to a shaikh eases my pain, just knowing that I am a small thing in a bigger world and my issues are smaller compared to others; this idea, for some reason, soothes people. I'm not against it in any way. (Woman, 18 years, South Asia, second generation)

Some participants used religious stories that highlight coping during times of crisis, as a way to encourage reliance on religion.

I think the imam can provide the precedence or guidance of things that could be… you know, because there were times when the prophet's life was difficult for example, the year of the sorrow, you know that. That was a difficult time, so they can provide guidance on what changes we can incorporate. (Woman, 48 years, South Asia, first generation)

##### Preference for Muslim Therapists

Most participants from both groups sought clarification about the distinction between a psychologist and a psychiatrist. However, second-generation participants demonstrated a slightly greater ability to articulate the differences between the two professions. They were also more aware that psychiatrists can prescribe medication, while also acknowledging the concerns related to its use.

I would first reach out to a psychologist so that I can talk out the issues that I’m having, to see if it's severe enough, whether I would need a vacation or prescription, and if they think that the issue that I'm dealing with is severe enough, then I would also reach out to a psychiatrist despite my concerns of medications’ side effects. But initially, I would go to a psychologist. (Woman, 19 years, South Asia, second generation)

By contrast, Abed, a first-generation man, was unsure about the distinction between the two professions:

All these are targeting the same. I'm not really sure [what's the difference]. I don’t know the exact term. (Man, 56 years, Middle East, first generation)

In addition, when opting to seek formal mental health assistance, the majority of participants across both generational groups expressed a preference for a Muslim therapist. Mesk voiced that she preferred having a Muslim therapist so that she does not end up paying for educating the therapists on her cultural and religious background in the first therapy sessions, and utilize the time spent in therapy on recovery with a therapist who fully understands her.

When I come to this stage of emotional distress in my life, I would say it would help if the psychiatrist or the psychologist was Muslim. It wouldn’t be like my first priority, but I think it would be something that I would consider, instead of having to educate my therapist on my background. (Woman, 19 years, Middle East, second generation)

## Discussion

This qualitative study explored intergenerational gaps (disparities and similarities) in seeking mental health help among first- and second-generation Muslims in California. Participants were Muslim Americans who resided in California. Our results revealed four major themes: the effects of acculturation, intentions, perceived behavioral control and pathways to seeking mental health help. These themes highlight both differences and similarities between the two groups.

### Differences

#### Differences in Mental Health Awareness, Normalization, and Knowledge

The second-generation group exhibited greater awareness, normalization, and acceptance of mental health challenges compared with the first-generation group. Second-generation participants were more comfortable discussing their mental health needs and seeking professional support, which was influenced by exposure at schools, higher education, workplaces, and media in California. Research shows that integrating a continuum component of mental health support directly into the school's system leads to improved social, emotional, behavioral, and academic outcomes (Hoover & Bostic, 2021). This environment helped normalize mental health care in their experiences, and fostering more favorable attitudes by emphasizing the benefits of seeking help, equating its importance to physical health.

Furthermore, the first generation continues to grapple with a cultural disjunction, feeling caught between their traditional cultural values—characterized by stigma and shame surrounding mental health—and the evolving perspectives of the dominant society. For first-generation participants, cultural beliefs about mental health care remained deeply ingrained, even for those who had studied or lived in the USA. This cultural tension made it difficult to fully embrace western approaches to mental health despite acknowledging their potential benefits. This struggle highlights the enduring impact of cultural heritage on attitudes toward mental health and the challenges faced in reconciling traditional beliefs with western approaches.

Understanding of mental health causality also differed between the two groups. Although many participants adopted a biopsychosocial perspective, some first-generation individuals associated mental health conditions with religious factors, such as a perceived weakness of faith. Some first-generation participants acknowledged personal resilience and emotional self-control as protective factors against distress but recognized the need for professional help in cases of chemical imbalances or challenges beyond their control. First-generation participants experienced acculturation stress, which shaped their beliefs and attitudes toward mental health. They were more influenced by social stigma, which affected their willingness to seek help. By contrast, the second-generation participants expressed more favorable attitudes, emphasizing the benefits of seeking professional mental health support. These findings were consistent with another study conducted in the UK, where younger generations had more favorable attitudes toward depression compared to older generations (McClelland et al., 2014).

#### Intentions

The two groups differed in their approach to seeking mental health help. The first generation tended to do so secretly, constrained by cultural norms and the stigma surrounding mental health. They expressed a desire to break free from these pressures, but often struggled to do so. By contrast, the second generation viewed seeking mental health support as one of many valid options, largely unaffected by cultural or societal stigma. For the second generation, mental health care was framed as a tool for personal growth and well-being, rather than a medicalized or stigmatized action.

#### Perceived Behavioral Control

Financial cost emerged as a significant barrier to accessing formal mental health services, particularly for second-generation participants who were mostly single and relied on parental support. High out-of-pocket fees may limit students’ ability to seek care independently, highlighting the need for affordable, accessible mental health options for young adults in this community. Conversely, first-generation participants, all of whom were married, described how their marital and family roles shaped their sense of control and support when considering mental health care.

### Similarities

Despite the diverse disparities between the two generational groups, they have also presented several similarities.

#### Preference for Informal Support

Both groups prioritized informal support systems, such as family, friends, and their inner societal community, before considering or seeking external/professional help. They emphasized the role of parents and elders in decision-making related to mental health and considered the family dynamic central to coping with challenges. It is common among individuals from collectivist backgrounds to first seek help from family members or religious figures before considering formal mental health services. These cultural and religious norms emphasize collective well-being and the importance of maintaining community-based solutions over individual therapy (Abideen & Abbas, 2021). This sense of collectivism fosters emotional security, closeness, and strong connections with significant figures (Rizkalla & Rahav, 2016). Kapadia et al. (2017) found that family was the primary support system for Pakistani women, unlike White women. Among Muslims, help-seeking is often described as a contextual process, where social networks offer support as an alternative to formal services, which is perceived as less judgmental (Badran et al., 2025a). Hashemi et al. (2019) found that among Middle Eastern migrants in Australia, ethnic acculturation was directly associated with psychological well-being because it provided individuals with a sense of belonging, stability, and relatedness due to shared collectivist values within their own ethnic group.

#### Importance of Religious Guidance and Preference for Muslim Therapists

Participants across generations valued religious coping mechanisms, such as consulting with Imams, reading the Quran, and drawing on religious stories for encouragement and solace. Religious practices, such as prayer and consultation with scholars, are often seen as valuable steps in managing mental health conditions, reflecting a strong cultural and religious influence*.* Both generations expressed a preference for Muslim therapists, highlighting the importance of shared cultural and religious understanding in fostering effective therapeutic relationships and outcomes. The impact of religion on individuals who have experienced difficult life events is a recurring topic in the literature, with ongoing debates about whether its influence is positive or negative (Cetty et al., 2022). Many studies highlight that religious practices often serve as a source of comfort, relief, a protective factor, and a sense of personal security (Abu-Raiya et al., 2011; Florez et al., 2018). Religion offers a framework for interpreting distress, which can help individuals reframe challenging situations in a more positive light, fostering resilience (Humam et al., 2023). In cultures where religion holds a dominant role, such as among Muslims, religious organizations/institutions and community events are crucial support systems (Badran et al., 2025a). These institutions not only provide spiritual guidance to Muslims, but they also serve as social networks that offer emotional, practical, and psychological support to individuals facing mental health challenges (Ali et al., 2022). The communal aspects of religious practices can create a sense of belonging and connectedness, which are essential for coping with distress and promoting well-being (Badran et al., 2025a). This notion is reinforced by a study on Muslim community members in California who expressed the need for developing support groups within their mosque space (W. M. Abu-Ras, 2003).

It is essential to acknowledge the diversity within Muslim communities, considering sociocultural backgrounds, country of origin, educational levels, economic status, degrees of religiosity, and migration/displacement experiences. Attitudes toward seeking mental health support may also shift over time as communities grow more familiar with mental health concepts and the services available to them. To address these needs, it is vital to ensure the availability of affordable, accessible, and culturally sensitive health and mental health services tailored to this population (Farrag & Hammad, 2005). Given that many younger adult Muslims reside with their families until attending distant universities, colleges, or marrying, the role of the family remains central in designing and implementing interventions for individuals experiencing mental health challenges. Furthermore, the preference for therapists who share similar religious beliefs and values underscores the importance of culturally sensitive care. Participants emphasized the importance of having their values and beliefs understood and respected during therapy, advocating for approaches that prioritized non-medicated treatments unless a diagnosis necessitated medication.

### Limitations and Implications

It is important to recognize that Muslim Americans are a diverse and heterogeneous population, which makes drawing meaningful comparisons across different subgroups very challenging. Thus, the findings of this study can only shed some light on the intergenerational disparities among Muslims who reside in California. In addition, factors such as the extent of religiosity and length of stay in the USA have been shown in other studies to influence the acculturation process. However, these variables were not included in the demographics of the current study, which might limit the ability to fully understand the nuances of acculturation within the studied population. Future studies may explore generational disparities across multiple US states.

## Conclusion

The study highlights important implications for research, practice, and policy. Culturally adapted interventions that integrate traditional beliefs with contemporary practices are needed, along with research on generational differences in mental health attitudes and informal support systems. mental health professionals should provide culturally sensitive care and actively engage with the family and religious support systems, especially with immigrant population. Educational outreach programs and culturally appropriate campaigns are essential. Policy efforts should ensure access to culturally appropriate services, allocating funding that mitigate social stigma.

## Data Availability

The data sets generated and analyzed during the current study are not publicly available due to concerns of privacy and confidentiality, but some material can be available from the corresponding author on reasonable request.
